# Gene Mapping of a Mutant Mungbean (*Vigna radiata* L.) Using New Molecular Markers Suggests a Gene Encoding a YUC4-like Protein Regulates the Chasmogamous Flower Trait

**DOI:** 10.3389/fpls.2016.00830

**Published:** 2016-06-10

**Authors:** Jingbin Chen, Prakit Somta, Xin Chen, Xiaoyan Cui, Xingxing Yuan, Peerasak Srinives

**Affiliations:** ^1^Institute of Vegetable Crops, Jiangsu Academy of Agricultural SciencesNanjing, China; ^2^Department of Agronomy, Faculty of Agriculture at Kamphaeng Saen, Kasetsart UniversityNakhon Pathom, Thailand

**Keywords:** mungbean, *Vigna radiata*, chasmogamy, outcrossing, gene mapping, hybrid development

## Abstract

Mungbean (*Vigna radiata* L.) is a cleistogamous plant in which flowers are pollinated before they open, which prevents yield improvements through heterosis. We previously generated a chasmogamous mutant (CM) mungbean in which open flowers are pollinated. In this study, we developed insertion/deletion (indel) markers based on the transcriptome differences between CM and Sulu-1 (i.e., normal flowering) plants. An F_2_ population derived from a cross between CM and Sulu-1 was used for gene mapping. Segregation analyses revealed that a single recessive gene regulates the production of chasmogamous flowers. Using newly developed indel and simple sequence repeat markers, the *cha* gene responsible for the chasmogamous flower trait was mapped to a 277.1-kb segment on chromosome 6. Twelve candidate genes were detected in this segment, including *Vradi06g12650*, which encodes a YUCCA family protein associated with floral development. A single base pair deletion producing a frame-shift mutation and a premature stop codon in *Vradi06g12650* was detected only in CM plants. This suggested that *Vradi06g12650* is a *cha* candidate gene. Our results provide important information for the molecular breeding of chasmogamous mungbean lines, which may serve as new genetic resources for hybrid cultivar development.

## Introduction

Exploiting heterosis is an effective way to increase crop yield. As an important legume in Asia, several studies have been conducted on mungbean (*Vigna radiata* L. Wilczek) to investigate the importance of heterosis for seed yield and other yield-related traits (Chen et al., 2003; Soehendi and Srinives, 2005; Sorajjapinun et al., 2012; Yashpal et al., 2015). However, a major obstacle to producing mungbean hybrid seeds is floral architecture. Mungbean plants have papilionaceous flowers with five differently shaped petals, including one standard petal, two wing petals, and two keel petals (Verdcourt, 1970). The anthers and stigmas are enclosed within the pocket-like keel petals, making mungbean a cleistogamous plant whose flowers are pollinated before they open. The natural outcrossing rate of cultivated mungbean is approximately 1.68% (Sangiri et al., 2007), which is too low for the commercial production of hybrid seeds. Some accessions of cleistogamous plants exhibit a specific floral architecture that promotes natural outcrossing. Studies in rice revealed that exerted stigmas and anthers increase the outcrossing rate and considerably help the production of hybrid seeds (Kato and Namai, 1987). Some mutant legume crops with exerted stigmas and anthers have been described (Dahiya et al., 1984; Pundir and Reddy, 1998; Cherian et al., 2006; Srinivasan and Gaur, 2012; Dheer et al., 2014). We previously identified a chasmogamous mutant (CM), which was generated in the mungbean accession V1197 by gamma irradiation (Sorajjapinun and Srinives, 2011). The outcrossing rate of CM plants increased to 9.6%, while outcrossing was undetectable in wild-type controls. Additionally, the yield and agronomic traits of CM plants differed from those of V1197 plants, with fewer pods per plant, seeds per pod, and yield per plant. Genetic analyses using F_1_, F_2_, and backcross populations from the cross between CM and V1197 plants revealed that the production of chasmogamous flowers is regulated by a single gene, *cha* (Sorajjapinun and Srinives, 2011). Thus, additional research focusing on the genetic characterization of this mutant mungbean should be conducted.

In terms of molecular genetics and genomics, mungbean has not been as extensively studied as other legumes, including soybean [*Glycine max* (L.) Merr.], common bean (*Phaseolus vulgaris* L.), cowpea [*V. unguiculata* (L.) Walp.], and chickpea (*Cicer arietinum* L.). DNA markers represent important tools for genetic analyses and the mapping of genes or quantitative trait loci. Many DNA markers, especially simple sequence repeats (SSRs), have been developed for mungbean (Gwag et al., 2007; Somta et al., 2008, 2009; Tangphatsornruang et al., 2009; Gupta et al., 2014; Chen et al., 2015) or introduced from other closely related species. These markers have been used to develop linkage maps and map quantitative trait loci associated with important mungbean traits (e.g., yield and resistance to biotic and abiotic stresses; Kasettranan et al., 2010; Chankaew et al., 2011, 2013; Isemura et al., 2012; Kajonphol et al., 2012; Prathet et al., 2012; Sompong et al., 2012; Kitsanachandee et al., 2013; Alam A.K.M.M. et al., 2014; Alam A.M. et al., 2014; Chotechung et al., 2016). However, most of these markers are monomorphic or weakly polymorphic. One of the most important advances in mungbean genomics research was the release of the VC1973A whole genome sequence by Kang et al. (2014). This sequence is relevant for research related to marker development, gene mapping, and gene function analyses.

The molecular mechanisms regulating floral development can be summarized using an “ABCE” model (Krizek and Fletcher, 2005). Additionally, some hormones (e.g., auxin) are important for floral development (Okada et al., 1991; Bennett et al., 1995; Przemeck et al., 1996; Galweiler et al., 1998; Aloni et al., 2006; Cheng et al., 2006). Some *Arabidopsis thaliana* mutants have abnormal flowers, and the genes responsible for this mutation have been identified as *pin1*, *pinoid*, *mp*, and *yuc* (Cheng and Zhao, 2007). The YUCCA (YUC) family consists of flavin monooxygenases (FMOs) related to the biosynthesis of indole-3-acetic acid. The FMOs are key enzymes that convert indole-3-pyruvic acid to indole-3-acetic acid by catalyzing the hydroxylation of the amino group of tryptophan, which is the rate-limiting step in tryptophan-dependent auxin biosynthesis (Zhao et al., 2001). There are 11 *YUC* genes in the *A. thaliana* genome (Zhao et al., 2001; Cheng et al., 2006, 2007). Double, triple, and quadruple mutants of some YUC family genes exhibit severe defects in floral patterns, vascular formations, and other developmental processes (Cheng et al., 2006). Therefore, *YUC* genes play important roles during floral formation and development, with implications for flower shape.

In this study, we mapped the *cha* gene regulating the production of chasmogamous flowers in CM plants using new insertion/deletion (indel) and SSR markers. We identified a gene encoding a YUC-like protein as a likely candidate for the *cha* gene.

## Materials and Methods

### Mapping Population and DNA Extraction

We previously identified a CM mungbean line in the M_2_ generation of accession V1197 following gamma irradiation. The CM plants lacked wing and keel petals, which exposed the stigmas and anthers (Sorajjapinun and Srinives, 2011). A stable CM line was selected from the M_3_ and M_4_ generations. A CM plant was pollinated using pollen from Sulu-1, which is a mungbean line with normal flowers. Three F_1_ plants were grown and the resulting flowers were morphologically analyzed. The hybrids were verified using two polymorphic indel markers (i.e., VRID001 and VRID002; Supplementary Table S1). Seeds from one F_1_ plant were then harvested to produce an F_2_ population consisting of 127 plants. The F_2_ plants and their parents were grown in a field at Kasetsart University, Kamphaeng Saen Campus, Nakhon Pathom, Thailand from May to July 2014. Flowers from each plant were individually examined to determine whether they were normal or chasmogamous.

Total genomic DNA was extracted from fresh leaf tissue of individual plants according to a slightly modified version of the method described by Lodhi et al. (1994). All DNA samples were diluted to 5 ng μl^-1^ according to lambda DNA, and analyzed by 1.0% agarose gel electrophoresis.

### Transcriptome Sequencing and Development of Molecular Markers

Total RNA was extracted from young flowers of CM and Sulu-1 mungbean plants using the EasyPure Plant RNA kit (Transgene Biotech, Beijing, China). The RNA samples were used to prepare libraries for sequencing by the Illumina HiSeq 2000 sequencer at the Beijing Genomics Institute (Shenzhen, China). The resulting sequences were assembled using the Trinity program (Grabherr et al., 2011). The Sulu-1 and CM transcriptome sequences were aligned using the NCBI BLAST+ 2.2.31 program with an *E*-value cutoff of 10.0. Sequences with indels that were 5 bp or larger were randomly chosen for marker development. Primers specific for the selected indels were designed using Primer3 (Untergasser et al., 2012) with the following criteria: primer length: 18–27 nucleotides; melting temperature: 50–65°C; GC content: 40–60%; and polymerase chain reaction (PCR) product size: 100–300 bp. Transcript sequences used for indel marker development were blasted against the mungbean whole genome sequence^1^ (Kang et al., 2014) to determine the physical locations of markers.

In addition to indel markers, we developed new SSR markers to fine map the *cha* locus. The mungbean chromosome 6 sequence was downloaded (Kang et al., 2014) and scanned for di-, tri-, and tetra-nucleotide repeats using SSR Hunter 1.3 (Li and Wan, 2005). Based on our initial mapping of the *cha* locus using indel markers, we focused on a 2.2-Mb genomic region of chromosome 6 (i.e., 29.9–32.1 Mb) carrying *cha*, and selected the SSRs therein. Primers for the SSRs were designed as described for the indel markers.

### Molecular Marker Analysis

The newly developed indel and SSR markers were used to detect polymorphisms between CM and Sulu-1 sequences. The PCR analyses were completed using a 10-μl solution containing 2 ng genomic DNA, 1x *Taq* buffer, 2 mM MgCl_2_, 0.2 mM dNTPs, 1 U *Taq* DNA polymerase (Thermo Scientific), and 0.5 μM forward and reverse primers. The PCR was conducted in an MJ Research PTC-200 Thermal Cycler (Bio-Rad) using the following program: 94°C for 3 min; 35 cycles of 94°C for 30 s, 55°C for 30 s, and 72°C for 30s; 72°C for 5 min. The amplicons were separated in a 6% denaturing polyacrylamide gel or a 3% agarose gel, and visualized with silver or ethidium bromide staining, respectively. The polymorphic markers were used to analyze the F_2_ population.

### Data Analysis and Gene Mapping

We counted the number of plants with normal and chasmogamous flowers, and analyzed the data using a χ^2^ test (Mather, 1951) to confirm the monogenic inheritance in CM plants as reported earlier by Sorajjapinun and Srinives (2011). Segregation data for the floral traits and DNA markers were used to construct a linkage map with MapMaker/EXP 3.0 (Lander et al., 1987). A minimum logarithm of odds value of 3.0 and maximum recombination frequency of 4.0 were used to group the markers. The genetic map distance was calculated using the Kosambi mapping function. Linkage groups were anchored to chromosomes by the physical location of markers. The map was drawn using MapChart 2.30 (Voorrips, 2002).

### Identifying and Sequencing the Candidate Gene

Based on the locations of the markers flanking *cha* on the linkage map, the predicted genes on the mungbean reference sequence (Kang et al., 2014) between the flanking markers were downloaded. Deduced protein sequences for these genes were subjected to a BLASTP search against the NCBI database to obtain information regarding their homologs and functions.

The following two pairs of primers were designed to amplify the genomic region containing a candidate gene responsible for the production of chasmogamous flowers: Seq-1-F (5′-AAGAACGAGGTTTGGCTTCA-3′)/Seq-1-R (5′-AAC TTGACCCACATCAAGGA-3′) and Seq-2-F (5′-GGAAAC ACAAATCACTATGGCA-3′)/Seq-2-R (5′-ATTGCATGTACA TGCCAGCTA-3′). The PCR was conducted using Platinum *Taq* DNA Polymerase High Fidelity (Invitrogen) following the manufacturer’s instructions with some modifications (i.e., annealing temperature of 58°C and elongation time of 90 s). The PCR products were separated by electrophoresis on 1% agarose gels stained with ethidium bromide. The DNA fragments of the expected size were purified using the E.Z.N.A Gel Extraction Kit (Omega Bio-tek), and sequenced using the 3730xl DNA Analyzer (Applied Biosystems). The deduced Vr06g12650 protein and 11 *A. thaliana* YUC proteins (i.e., AT4G32540.1, AT4G13260.1, AT1G04610.1, AT5G11320.1, AT5G43890.1, AT5G25620.2, AT2G33230.1, AT4G28720.1, AT1G04180.1, and AT1G48910.1) obtained from The *Arabidopsis* Information Resource^2^ database were used to construct a phylogenetic tree using the “One Click” mode and LRT statistical test of Phylogeny.fr (Dereeper et al., 2010).

## Results

### Morphological Features of the Chasmogamous Mutant Mungbean and Inheritance of the Floral Trait

**FIGURE 1:**
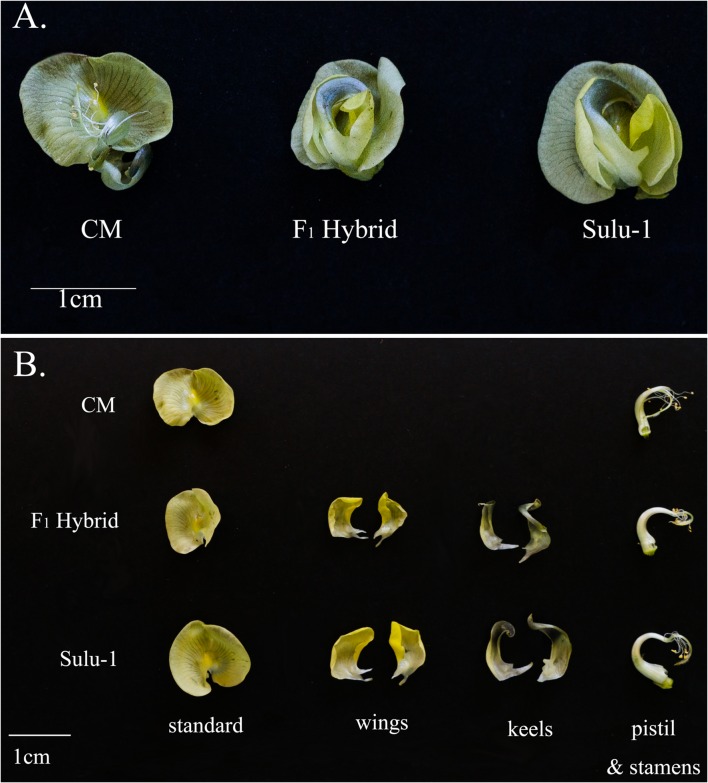
**Floral morphology of CM and Sulu-1 plants as well as their F_1_ hybrids.** Whole flower **(A)** and individual flower parts **(B)**.

The floral architecture of CM plants differed from that of Sulu-1 plants. The CM flowers were missing the wing and keel petals (**Figure 1**). When the CM and Sulu-1 plants were crossed, the F_1_ hybrids had normal flowers (**Figure 1**), which suggested a recessive gene (or genes) regulated the production of chasmogamous flowers in CM plants.

We determined the segregation ratio of the chasmogamous flower trait in F_2_ plants. Out of 127 plants, 96 had normal flowers, while 31 had chasmogamous flowers. This segregation corresponded with a 3:1 ratio (χ^2^ = 0.02, *P* = 0.88), indicating that a single recessive gene mediated the chasmogamous flower trait in CM plants. This confirmed the results of the study by Sorajjapinun and Srinives (2011).

### Development of Indel Markers

**FIGURE 2:**
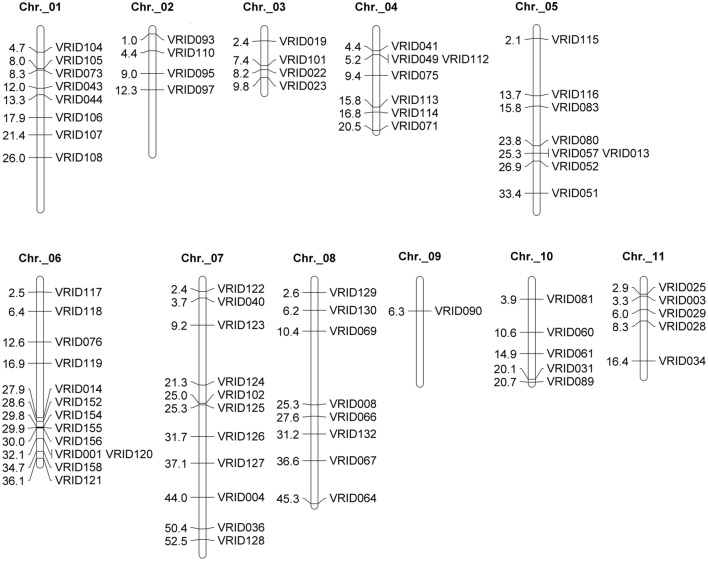
**Physical positions on the mungbean chromosome of the indel markers polymorphic between CM and Sulu-1 plants.** Unit: mega base pair (Mbp). Ten markers located on scaffolds are not shown.

To map the gene responsible for the production of chasmogamous flowers, indel markers were developed using the transcriptomes of the Sulu-1 and CM parents. We randomly selected 140 transcript sequences carrying indels to develop markers, and determined that 84 of them (i.e., 57.1%) were polymorphic between the parents (Supplementary Table S1). All of the markers were co-dominant, and able to distinguish between the parents and hybrid progenies. Seventy-four markers were localized to 11 chromosomes by aligning their related transcripts with the whole mungbean genome sequence (**Figure 2**). Another 10 markers were located on scaffolds that could not be assembled on chromosomes.

### Genetic Mapping of the *cha* Gene

**FIGURE 3:**
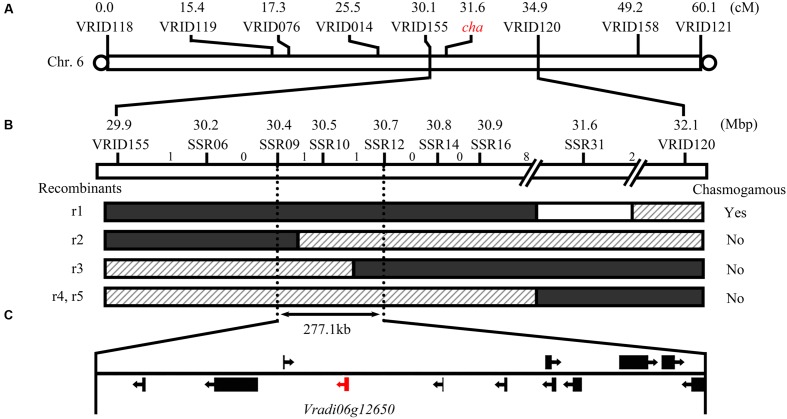
**Mapping of the *cha* gene regulating the production of chasmogamous flowers in CM plants.** Location of *cha* **(A)** and recombinant events on chromosome 6 **(B)**. White, shadow, and black regions in the recombinants indicate homozygous regions for the Sulu-1 allele, and heterozygous and homozygous regions for the CM allele, respectively. The number of recombinant events between adjacent markers are indicated above the bar. Another five recombinants between the homozygous Sulu-1 genotype and heterozygous genotype are not presented because all of the plants had normal flowers. The 12 predicted genes in the candidate gene region are indicated **(C)**.

Linkage analysis of the polymorphic indel markers and phenotyping of the flowers were completed using 127 plants from the F_2_ population. The target *cha* gene was mapped to chromosome 6 between markers VRID155 and VRID120 at a distance of 1.6 and 3.4 cM, respectively (**Figure 3A**). To locate *cha* more precisely, seven polymorphic SSR markers were developed in the target interval by screening SSR motifs in the reference genome sequence (Supplementary Table S2). Eight recombinants were identified in the interval between VRID115 and VRID120. By associating the marker genotypes with floral phenotypes, the 60 and 38 recombinants restricted *cha* a segment between markers SSR09 and SSR12. These two markers were 277.1 kb apart, and were located at 30.40 and 30.68 Mb of chromosome 6, respectively (**Figure 3B**). Based on the mungbean whole genome sequence, 12 candidate genes (i.e., *Vradi06g12620* to *Vradi06g12730*; Supplementary Table S3) were detected in this region.

### Function Prediction and Sequencing of *cha* Candidate Genes

**FIGURE 4:**
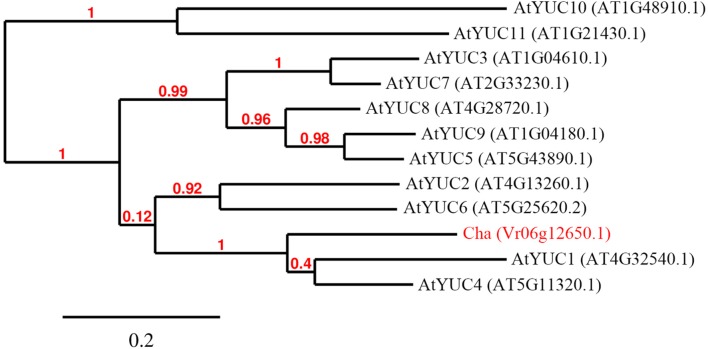
**Phylogenetic tree of *Arabidopsis thaliana* YUCCA and Cha proteins.** Sequences of 11 *A. thaliana* YUCCA proteins and the deduced mungbean Vr06g12650.1 protein were used to generate the phylogenetic tree.

**FIGURE 5:**
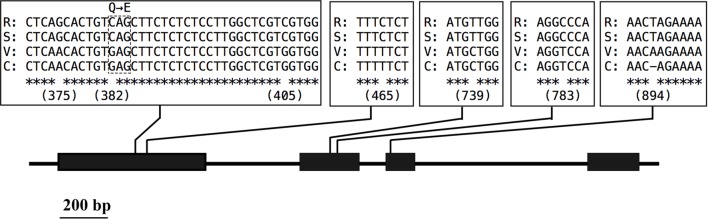
**Variability in the *Vradi06g12650* open reading frame.** Consensus sequences are marked by asterisks. Numbers under variant loci indicate the position (in bp) on the open reading frame. The R, S, V, and C correspond to V1973A (reference sequence), Sulu-1, V1197, and CM, respectively.

**FIGURE 6:**
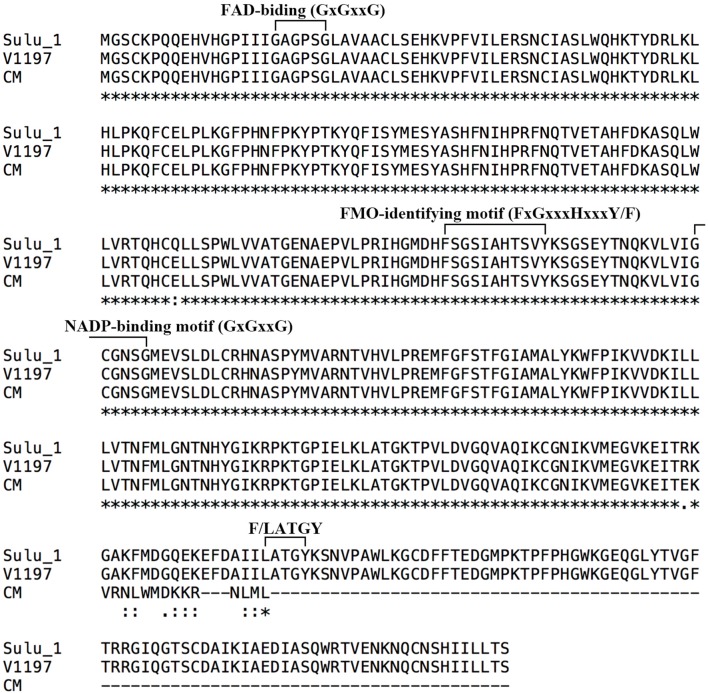
**Alignment of the deduced Cha protein sequences from Sulu-1, V1197, and CM plants by ClustalX.** Conserved flavin monooxygenase motifs are indicated (on top). Asterisk indicates conserved amino acid.

To predict the functions of the 12 detected *cha* candidate genes, the putative protein sequences encoded by these genes were used as queries to search the NCBI database. Their predicted functions are listed in Table S3. *Vradi06g12650*, which encoded a YUC homolog (**Figure 3**) related to the auxin biosynthesis pathway and floral development, was considered the most likely *cha* gene. A BLASTP search revealed that the YUC protein encoded by *Vradi06g12650* was most similar to the *A. thaliana* proteins AtYUC4 (identity: 68.13%) and AtYUC1 (identity: 62.32%). A protein sequence-based phylogenetic analysis involving Vradi06g12650 and 11 *A. thaliana* YUC proteins also revealed a close relationship between Vradi06g12650 and AtYUC1/AtYUC4 (**Figure 4**). We sequenced the *Vradi06g12650* coding domain in Sulu-1, CM, and wild-type V1197 mungbean plants. Comparisons among the resulting sequences and the corresponding reference sequence (i.e., from VC1973A) revealed six single nucleotide polymorphisms (SNPs) between the CM and Sulu-1 genes (**Figure 5**). The SNP at the 382-bp position led to an amino acid substitution from glutamine to glutamic acid, while the other SNPs resulted in non-sense mutations (**Figures 5** and **6**). The six SNPs were not detected between the CM and V1197 sequences. However, a 1-bp deletion at the 894-bp position was observed in the CM *Vradi06g12650* sequence (**Figure 5**). This deletion produced a frame-shift mutation in the 3′-terminus, resulting in a shorter CM Vradi06g12650 protein with a different C-terminus compared with the corresponding wild-type V1197, Sulu-1, and VC1973A proteins (**Figure 6**). Additionally, this deletion was detected in a sequence (i.e., Unigene0038420) from the CM transcriptome (Supplementary Data 1). These results suggested that the 1-bp deletion in *Vradi06g12650* is responsible for the production of chasmogamous flowers in CM plants.

## Discussion

### Advantages of Developing Markers from Transcriptomes and the Utility of *cha* for Mungbean Improvement

Mungbean is not a model plant. Therefore, its molecular genetics and genome have not been as comprehensively studied as in many other crops. The available molecular markers are insufficient for genetics research and mungbean breeding programs. Additionally, some reports have indicated that the polymorphic information content of mungbean SSR markers is low (Tangphatsornruang et al., 2009; Chen et al., 2015). Thus, it is important to develop more mungbean genomic resources. In this study, we developed indel markers by comparing the transcriptomes of two mungbean accessions, which resulted in the detection of several polymorphic markers between the parents of the mapping population. The differences in indel sizes between Sulu-1 and CM plants enabled the indels to be distinguished by PCR followed by 3% agarose gel electrophoresis analysis (**Supplementary Figure S1**), which is easier to complete than polyacrylamide gel electrophoresis. This resulted in a more efficient genetic mapping procedure.

With the newly developed markers, the *cha* gene responsible for the production of chasmogamous flowers in mungbean was mapped to chromosome 6. Several newly developed markers were identified closely linked to the target gene. Our data revealed that the marker SSR10 was completely linked to *cha*. However, this finding is based on a relatively small population of 127 F_2_ plants. Because the CM floral phenotype was observed to be regulated by a single recessive gene, these linked markers may be useful for the marker-assisted selection of mungbean plants producing chasmogamous flowers.

### Functions of the *cha* Candidate Gene

*Vradi06g12650* is the most likely *cha* gene. This gene putatively encodes a YUC homolog involved in auxin biosynthesis and floral development (Zhao et al., 2001; Cheng et al., 2006). The YUC proteins constitute a family of FMOs containing several conserved sequence motifs, including the FAD-binding motif, FMO-identifying motif, NADPH-binding motif, and F/LATGY motif (Schlaich, 2007). The protein encoded by *Vradi06g12650* is highly similar to *A. thaliana* YUC4 (**Supplementary Figure S2**). The CM *Vradi06g12650* coding sequence differed from that of the wild-type V1197 by a 1-bp deletion at the 894-bp position. This deletion was only detected in CM plants, and results in a frame-shift of the coding sequence leading to the absence of the LATGY motif in the C-terminus of the predicted YUC protein (**Figure 6**). There are 11 *A. thaliana* genes encoding YUC proteins, suggesting there may be some functional redundancy among these proteins. Mutational inactivation of a single *YUC* family gene in *A. thaliana* caused no obvious developmental defects, while a double *yuc1 yuc4* mutant and a quadruple *yuc1 yuc4 yuc10 yuc11* mutant exhibited severe defects in the formation of floral organs (Zhao et al., 2001; Cheng et al., 2006). These findings differed from our observation that a defect in a single *YUC4*-like gene causes a dramatic morphological abnormality in mungbean floral organs. However, it is worth noting that the mutation detected in our study produced an immature YUC4-like protein lacking the F/LATGY motif, which is highly conserved among YUC proteins (**Supplementary Figure S2**). Therefore, this defective YUC4-like protein may be responsible for the abnormal floral development in CM plants. Additional studies are required to characterize how *cha* affects the production of chasmogamous flowers in mungbean.

## Author Contributions

JC designed the InDel and SSR markers and prepared the manuscript. PS performed gene mapping and reviewed the manuscript. XChen involved in bioinformatics analysis and reviewed the manuscript. XCui sequenced the candidate gene. XY conducted the hybrids and developed the populations. PS designed the study and refined the manuscript.

## Conflict of Interest Statement

The authors declare that the research was conducted in the absence of any commercial or financial relationships that could be construed as a potential conflict of interest.

## Footnotes

1 http://plantgenomics.snu.ac.kr/mediawiki-1.21.3/index.php/Main_Page

2 http://arabidopsis.org

## Supplementary Material

**FIGURE S1 | Polymorphic InDel markers on 3% agarose gel.** Polymorphisms of 48 markers with large variance can be detected by 3% agarose gel. For each marker, bands from left to right are the PCR product from Sulu-1, CM and an F1 hybrid.

**FIGURE S2 | Protein sequence alignment of arabidopsis YUC1, YUC4 and Cha candidate.** Sequences of AtYUC1 (AT4G32540.1) and AtYUC4 (AT5G11320.1) were downloaded from NCBI. Cha is the deduced protein of mungbean Vr06g12650.1. Alignment was performed by ClustalX, consensus sequence was marked by asterisks. Conserved motifs of FMOs were mark on the top of sequences.
