# Supplementary figures and images for: Gene Mapping of a Mutant Mungbean (Vigna radiata L.) Using New Molecular Markers Suggests a Gene Encoding a YUC4-like Protein Regulates the Chasmogamous Flower Trait
Source: Front Plant Sci. 2016 Jun 10;7:830. doi: 10.3389/fpls.2016.00830 (PMC4901043; doi:10.3389/fpls.2016.00830)

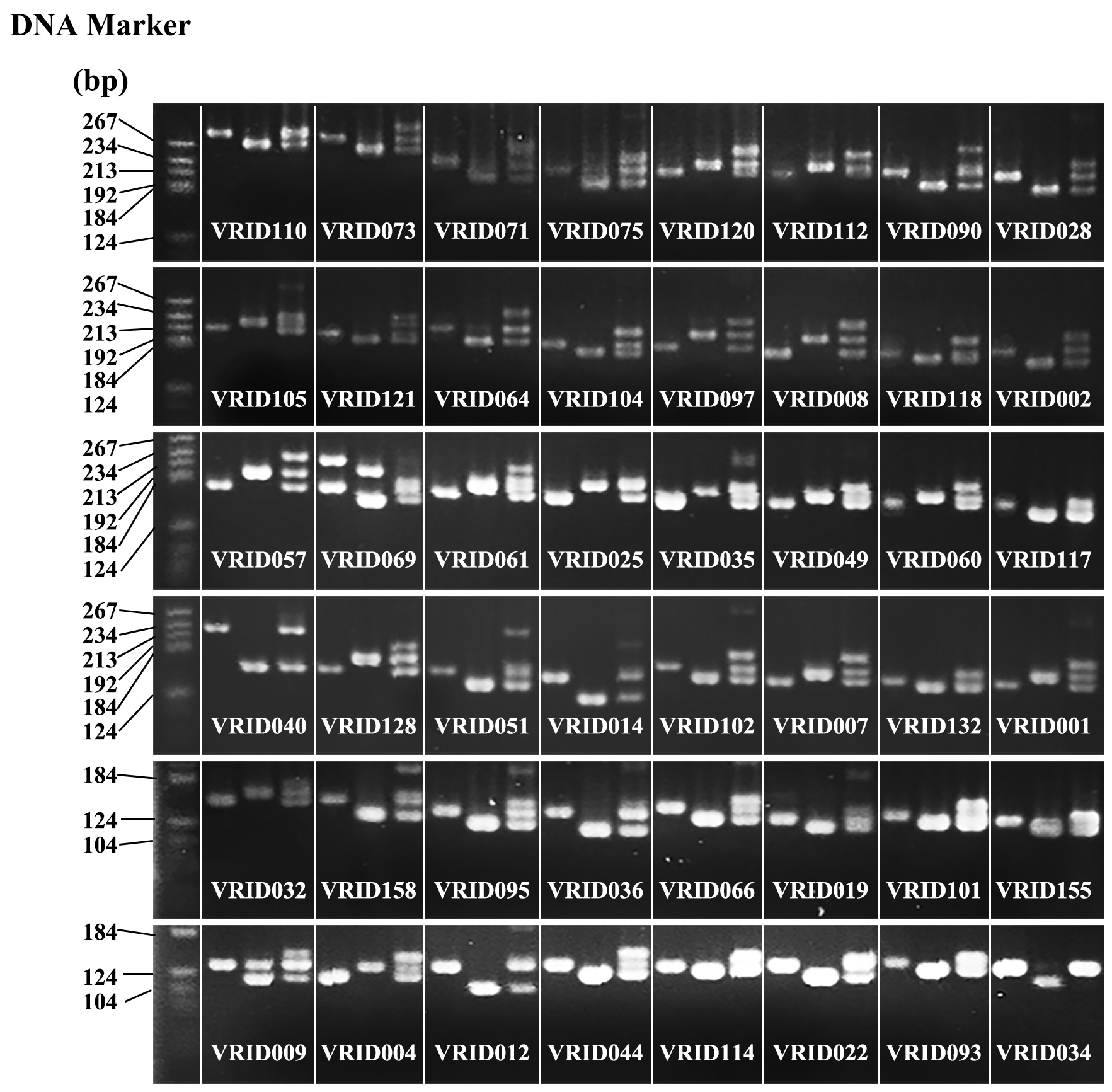

Supplement: Supplementary file 1 [file Image_1.JPG]

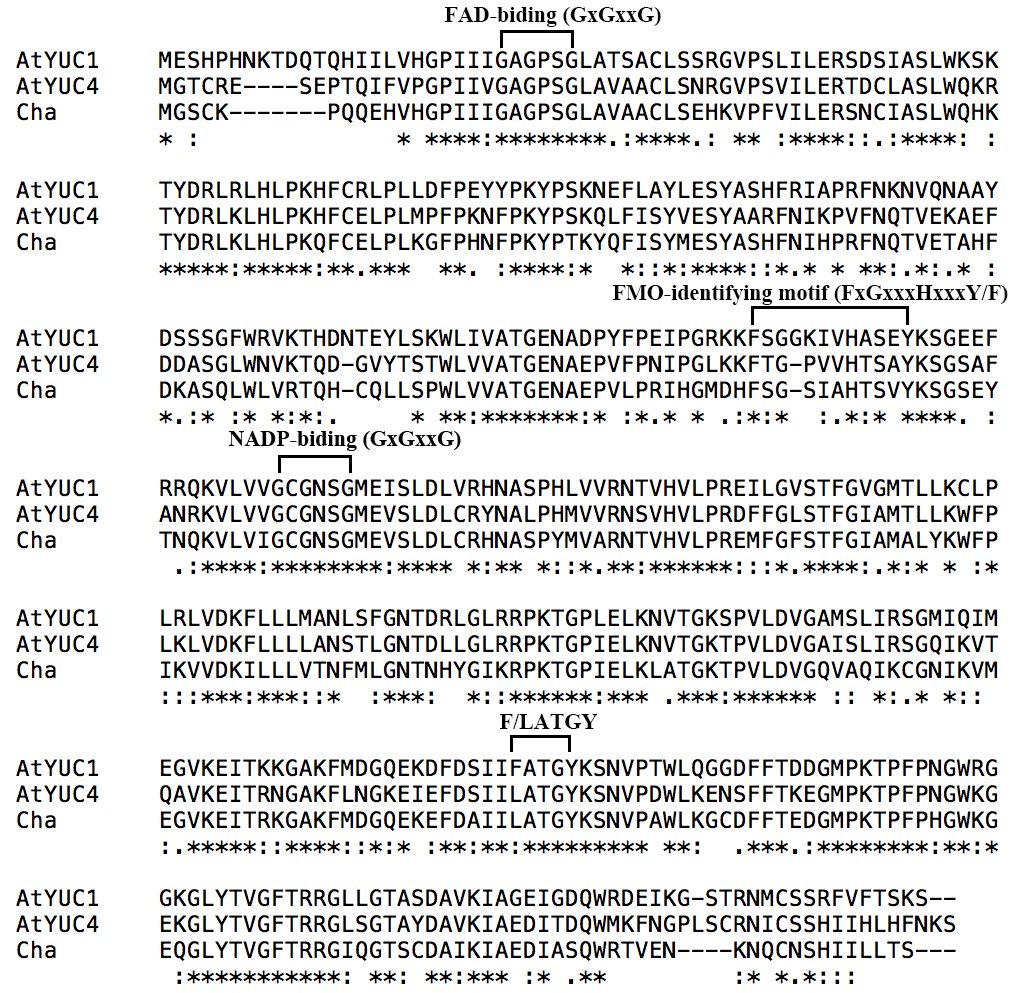

Supplement: Supplementary file 2 [file Image_2.JPG]
