# Supplementary material for: Gene Mapping of a Mutant Mungbean (Vigna radiata L.) Using New Molecular Markers Suggests a Gene Encoding a YUC4-like Protein Regulates the Chasmogamous Flower Trait
Source: Front Plant Sci. 2016 Jun 10;7:830. doi: 10.3389/fpls.2016.00830 (PMC4901043; doi:10.3389/fpls.2016.00830)
Supplement: Supplementary file 6 [file Data_Sheet_1.PDF]

CLUSTAL 2.1 multiple sequence alignment

```
Sulu_1              ------------------------------------------------------------
V1197               ------------------------------------------------------------
CM                  ------------------------------------------------------------
Unigene0038420      TTCAAAACTCTCACCTTTCCTTCTTGTTTTGTTCTTTCTCTTTTTCTCTCACACAAATAC


Sulu_1              --------------ATGGGTTCTTGCAAACCCCAACAAGAACATGTTCATGGACCTATCA
V1197               --------------ATGGGTTCTTGCAAACCCCAACAAGAACATGTTCATGGACCTATCA
CM                  --------------ATGGGTTCTTGCAAACCCCAACAAGAACATGTTCATGGACCTATCA
Unigene0038420      ACCTATCTATCTACATGGGTTCTTGCAAACCCCAACAAGAACATGTTCATGGACCTATCA
                                  **********************************************

Sulu_1              TCATAGGTGCCGGTCCTTCAGGCCTAGCCGTGGCTGCGTGTCTCTCGGAGCACAAAGTCC
V1197               TCATAGGTGCCGGTCCTTCAGGCCTAGCCGTGGCTGCGTGTCTCTCGGAGCACAAAGTCC
CM                  TCATAGGTGCCGGTCCTTCAGGCCTAGCCGTGGCTGCGTGTCTCTCGGAGCACAAAGTCC
Unigene0038420      TCATAGGTGCCGGTCCTTCAGGCCTAGCCGTGGCTGCGTGTCTCTCGGAGCACAAAGTCC
                    ************************************************************

Sulu_1              CTTTCGTGATTCTTGAGAGAAGCAACTGCATAGCCTCTCTTTGGCAACACAAAACCTACG
V1197               CTTTCGTGATTCTTGAGAGAAGCAACTGCATAGCCTCTCTTTGGCAACACAAAACCTACG
CM                  CTTTCGTGATTCTTGAGAGAAGCAACTGCATAGCCTCTCTTTGGCAACACAAAACCTACG
Unigene0038420      CTTTCGTGATTCTTGAGAGAAGCAACTGCATAGCCTCTCTTTGGCAACACAAAACCTACG
                    ************************************************************

Sulu_1              ACCGTCTCAAACTCCACCTCCCAAAGCAGTTCTGCGAGCTTCCCTTGAAAGGTTTTCCCC
V1197               ACCGTCTCAAACTCCACCTCCCAAAGCAGTTCTGCGAGCTTCCCTTGAAAGGTTTTCCCC
CM                  ACCGTCTCAAACTCCACCTCCCAAAGCAGTTCTGCGAGCTTCCCTTGAAAGGTTTTCCCC
Unigene0038420      ACCGTCTCAAACTCCACCTCCCAAAGCAGTTCTGCGAGCTTCCCTTGAAAGGTTTTCCCC
                    ************************************************************

Sulu_1              ACAACTTCCCCAAGTACCCCACAAAGTACCAGTTCATATCCTACATGGAGTCCTACGCCT
V1197               ACAACTTCCCCAAGTACCCCACAAAGTACCAGTTCATATCCTACATGGAGTCCTACGCCT
CM                  ACAACTTCCCCAAGTACCCCACAAAGTACCAGTTCATATCCTACATGGAGTCCTACGCCT
Unigene0038420      ACAACTTCCCCAAGTACCCCACAAAGTACCAGTTCATATCCTACATGGAGTCCTACGCCT
                    ************************************************************

Sulu_1              CACACTTCAACATCCACCCCAGGTTCAACCAAACAGTCGAAACTGCTCACTTTGATAAAG
V1197               CACACTTCAACATCCACCCCAGGTTCAACCAAACAGTCGAAACTGCTCACTTTGATAAAG
CM                  CACACTTCAACATCCACCCCAGGTTCAACCAAACAGTCGAAACTGCTCACTTTGATAAAG
Unigene0038420      CACACTTCAACATCCACCCCAGGTTCAACCAAACAGTCGAAACTGCTCACTTTGATAAAG
                    ************************************************************
```

```
Sulu_1              CCTCTCAGCTTTGGCTCGTTAGGACTCAGCACTGTCAGCTTCTCTCTCCTTGGCTCGTCG
V1197               CCTCTCAGCTTTGGCTCGTTAGGACTCAACACTGTGAGCTTCTCTCTCCTTGGCTCGTGG
CM                  CCTCTCAGCTTTGGCTCGTTAGGACTCAACACTGTGAGCTTCTCTCTCCTTGGCTCGTGG
Unigene0038420      CCTCTCAGCTTTGGCTCGTTAGGACTCAACACTGTGAGCTTCTCTCTCCTTGGCTCGTGG
                    **************************** ****** ********************** *

Sulu_1              TGGCCACCGGGGAGAATGCTGAGCCTGTGCTTCCTAGAATTCATGGCATGGACCATTTCT
V1197               TGGCCACCGGGGAGAATGCTGAGCCTGTGCTTCCTAGAATTCATGGCATGGACCATTTTT
CM                  TGGCCACCGGGGAGAATGCTGAGCCTGTGCTTCCTAGAATTCATGGCATGGACCATTTTT
Unigene0038420      TGGCCACCGGGGAGAATGCTGAGCCTGTGCTTCCTAGAATTCATGGCATGGACCATTTTT
                    ********************************************************** *

Sulu_1              CTGGCTCCATTGCTCACACCAGTGTCTACAAGTCTGGCTCTGAGTACACAAACCAGAAGG
V1197               CTGGCTCCATTGCTCACACCAGTGTCTACAAGTCTGGCTCTGAGTACACAAACCAGAAGG
CM                  CTGGCTCCATTGCTCACACCAGTGTCTACAAGTCTGGCTCTGAGTACACAAACCAGAAGG
Unigene0038420      CTGGCTCCATTGCTCACACCAGTGTCTACAAGTCTGGCTCTGAGTACACAAACCAGAAGG
                    ************************************************************

Sulu_1              TTCTCGTCATTGGCTGTGGCAATTCAGGAATGGAAGTTAGCTTAGACCTTTGCAGACACA
V1197               TTCTCGTCATTGGCTGTGGCAATTCAGGAATGGAAGTTAGCTTAGACCTTTGCAGACACA
CM                  TTCTCGTCATTGGCTGTGGCAATTCAGGAATGGAAGTTAGCTTAGACCTTTGCAGACACA
Unigene0038420      TTCTCGTCATTGGCTGTGGCAATTCAGGAATGGAAGTTAGCTTAGACCTTTGCAGACACA
                    ************************************************************

Sulu_1              ATGCCTCCCCTTACATGGTTGCAAGGAACACAGTGCATGTCCTTCCTAGGGAGATGTTTG
V1197               ATGCCTCCCCTTACATGGTTGCAAGGAACACAGTGCATGTCCTTCCTAGGGAGATGTTTG
CM                  ATGCCTCCCCTTACATGGTTGCAAGGAACACAGTGCATGTCCTTCCTAGGGAGATGTTTG
Unigene0038420      ATGCCTCCCCTTACATGGTTGCAAGGAACACAGTGCATGTCCTTCCTAGGGAGATGTTTG
                    ************************************************************

Sulu_1              GCTTCTCAACTTTTGGCATAGCCATGGCTCTTTACAAGTGGTTTCCCATCAAAGTTGTAG
V1197               GCTTCTCAACTTTTGGCATAGCCATGGCTCTTTACAAGTGGTTTCCCATCAAAGTTGTAG
CM                  GCTTCTCAACTTTTGGCATAGCCATGGCTCTTTACAAGTGGTTTCCCATCAAAGTTGTAG
Unigene0038420      GCTTCTCAACTTTTGGCATAGCCATGGCTCTTTACAAGTGGTTTCCCATCAAAGTTGTAG
                    ************************************************************

Sulu_1              ACAAAATTCTCTTACTTGTGACCAACTTCATGTTGGGAAACACAAATCACTATGGCATCA
V1197               ACAAAATTCTCTTACTTGTGACCAACTTCATGCTGGGAAACACAAATCACTATGGCATCA
CM                  ACAAAATTCTCTTACTTGTGACCAACTTCATGCTGGGAAACACAAATCACTATGGCATCA
Unigene0038420      ACAAAATTCTCTTACTTGTGACCAACTTCATGCTGGGAAACACAAATCACTATGGCATCA
                    ******************************** ***************************

Sulu_1              AAAGGCCTAAAACAGGCCCAATAGAGCTGAAACTAGCCACAGGGAAAACCCCAGTCCTTG
```

```
V1197              AAAGGCCTAAAACAGGTCCAATAGAGCTGAAACTAGCCACAGGGAAAACCCCAGTCCTTG
CM                 AAAGGCCTAAAACAGGTCCAATAGAGCTGAAACTAGCCACAGGGAAAACCCCAGTCCTTG
Unigene0038420     AAAGGCCTAAAACAGGTCCAATAGAGCTGAAACTAGCCACAGGGAAAACCCCAGTCCTTG
                   **************** *******************************************

Sulu_1             ATGTGGGTCAAGTTGCACAGATCAAATGTGGCAACATAAAGGTGATGGAAGGTGTGAAGG
V1197              ATGTGGGTCAAGTTGCACAGATCAAATGTGGCAACATAAAGGTGATGGAAGGTGTGAAGG
CM                 ATGTGGGTCAAGTTGCACAGATCAAATGTGGCAACATAAAGGTGATGGAAGGTGTGAAGG
Unigene0038420     ATGTGGGTCAAGTTGCACAGATCAAATGTGGCAACATAAAGGTGATGGAAGGTGTGAAGG
                   ************************************************************

Sulu_1             AGATAACTAGAAAAGGTGCGAAATTTATGGATGGACAAGAAAAGGAATTTGATGCTATAA
V1197              AGATAACAAGAAAAGGTGCGAAATTTATGGATGGACAAGAAAAGGAATTTGATGCTATAA
CM                 AGATAAC-AGAAAAGGTGCGAAATTTATGGATGGACAAGAAAAGGAATTTGATGCTATAA
Unigene0038420     AGATAAC-AGAAAAGGTGCGAAATTTATGGATGGACAAGAAAAGGAATTTGATGCTATAA
                   ******* ****************************************************

Sulu_1             TATTGGCAACAGGGTACAAGAGCAACGTGCCTGCTTGGCTTAAGGGTTGTGATTTTTTCA
V1197              TATTGGCAACAGGGTACAAGAGCAACGTGCCTGCTTGGCTTAAGGGTTGTGATTTTTTCA
CM                 TATTGGCAACAGGGTACAAGAGCAACGTGCCTGCTTGGCTTAAGGGTTGTGATTTTTTCA
Unigene0038420     TATTGGCAACAGGGTACAAGAGCAACGTGCCTGCTTGGCTTAAGGGTTGTGATTTTT---
                   *********************************************************

Sulu_1             CTGAGGATGGAATGCCGAAAACACCCTTTCCCCATGGGTGGAAAGGGGAGCAGGGATTGT
V1197              CTGAGGATGGAATGCCGAAAACACCCTTTCCCCATGGGTGGAAAGGGGAGCAGGGATTGT
CM                 CTGAGGATGGAATGCCGAAAACACCCTTTCCCCATGGGTGGAAAGGGGAGCAGGGATTGT
Unigene0038420     ------------------------------------------------------------

Sulu_1             ATACGGTGGGGTTCACCAGAAGAGGCATTCAAGGAACATCTTGTGATGCAATCAAGATCG
V1197              ATACGGTGGGGTTCACCAGAAGAGGCATTCAAGGAACATCTTGTGATGCAATCAAGATCG
CM                 ATACGGTGGGGTTCACCAGAAGAGGCATTCAAGGAACATCTTGTGATGCAATCAAGATCG
Unigene0038420     ------------------------------------------------------------

Sulu_1             CTGAAGACATAGCCTCGCAGTGGAGAACCGTAGAGAACAAGAATCAATGCAATTCACATA
V1197              CTGAAGACATAGCCTCGCAGTGGAGAACCGTAGAGAACAAGAATCAATGCAATTCACATA
CM                 CTGAAGACATAGCCTCGCAGTGGAGAACCGTAGAGAACAAGAATCAATGCAATTCACATA
Unigene0038420     ------------------------------------------------------------

Sulu_1             TCATCCTTCTCACTTCATAA
V1197              TCATCCTTCTCACTTCATAA
CM                 TCATCCTTCTCACTTCATAA
```

Unigene0038420 --------------------
